# Supplementary material for: Chromothripsis during telomere crisis is independent of NHEJ, and consistent with a replicative origin
Source: Genome Res. 2019 May;29(5):737–49. doi: 10.1101/gr.240705.118 (PMC6499312; doi:10.1101/gr.240705.118)
Supplement: Supplemental Material [file supp_gr.240705.118_Supplemental_file_1.zip › contigs/annotated_contigs/DB54/contig.2.DB54_length_361_mean_cov_9.41828254848.docx]

**DB54_length_361_mean_cov_9.41828254848**

```
AAGTGAA|AAAATTAAAAGGCAATATAATTTACAGAACATCTTAAATACTCGGGTATACATCTAACAAAAACTTGTATAGTGTCTGTTT
        >chr7:1861828-1862079 + E=8e-137 p=0e+00
GCAGAAAGTTAGGAAATACTGATGAAAGGAATCGAAAGGAATCAAAAGAATGTTTGTATAAATAGATACAATGTTTATGAACTGGAAGA

CTTATAGTAAATCAATTTTTCCAAAATTGATCTTTGGTTTTAAGGTAATTGCTGTCCAAACCTCAGCATATATAT|ATATAT|ATATAT
                                                                            >chr9:17352
ATATATATATATATATATTTTTTTTTTTTTGTATG|AGGGTTTTTTTTTGTGTCTCTTGTTTTGGTGTAGGTGTGTGGTTTTTGGTTTC
387-17352434 + E=8e-11
TTTATACTC
```
